# The use of ELISAs for monitoring exposure of pig herds to *Brachyspira hyodysenteriae*

**DOI:** 10.1186/1746-6148-8-6

**Published:** 2012-01-17

**Authors:** Yong Song, Barbara Frey, David J Hampson

**Affiliations:** 1 Animal Research Institute, School of Veterinary and Biomedical Science, Murdoch University, Murdoch, Western Australia 6150, Australia; 2 Consistent Pork, PO Box 6901, East Perth, Western Australia 6892, Australia; 3 Faculty of Medicine, Dentistry and Health Sciences, The University of Western Australia, M550, 35 Stirling Highway, Crawley, 6009, Western Australia, Australia

**Keywords:** Antibody profiles, *Brachyspira hyodysenteriae*, ELISA, Meat juice, Swine dysentery

## Abstract

**Background:**

Swine dysentery (SD), a mucohaemorrhagic diarrhoeal disease of pigs, results from infection of the large intestine with the spirochaete *Brachyspira hyodysenteriae*. ELISA systems using whole spirochaete cells (WC) and the *B. hyodysenteriae *outer membrane lipoprotein Bhlp29.7 previously have been established as potential diagnostic tools for SD. However, their true value in identifying infected herds remains unclear. The present study aimed to compare the performance of whole-cell and Bhlp29.7 based ELISAs in detecting specific immunoglobulin class IgG and IgM to *B. hyodysenteriae *in growing pigs, and additionally evaluated whether meat juice could serve as a source of specific antibodies.

**Results:**

Levels of circulating IgG and IgM reacting with WC spirochaete preparations and recombinant Bhlp29.7 peaked 4-6 weeks post-infection in the experimentally challenged pigs, and remained elevated in the present study. In a cohort of pigs on an infected farm levels of antibody directed against both antigens showed a progressive increase with time. However, other than for the level of IgG against WC antigen, a significant increase in antibody levels also was observed in a cohort of pigs on a non-infected farm. In addition, assays using meat juice had 100% specificity and equivalent sensitivity to those based on serum, and likewise the best performance was achieved using the WC IgG ELISA.

**Conclusions:**

IgG ELISAs using either WC or Bhlp29.7 as plate-coating antigens were shown to be useful for monitoring the dynamics of *B. hyodysenteriae *infection in grower pigs. Of the two antigens, the WC preparation tended to give better discrimination between pigs from infected and non-infected farms. Testing of meat juice was shown to have potential for identifying infected herds.

## Background

Swine dysentery (SD) is a mucohaemorrhagic colitis of pigs resulting from infection of the large intestine with the anaerobic spirochaete *Brachyspira hyodysenteriae *[1]. The disease has a worldwide distribution and causes significant economic loss. Successful control programs for SD rely on accurate, cost-effective and timely detection of infected herds and animals. Diagnosis is usually based on clinical signs and detection of the causative spirochaete by culture and/or PCR [2-4]. Although not widely used, serological tests measuring serum immunoglobulins specific to *B. hyodysenteriae *have potential to be used at the herd level for routine surveillance [5]. ELISA systems using whole spirochaete cells [6,7], lipopolysaccharide (LPS) [8], and the *B. hyodysenteriae *outer membrane lipoprotein Bhlp29.7 [9] as antigens all have been described for this purpose. Elevated antibody levels have been detected in pigs for up to 150 days following experimental infection [10], and hence sera obtained from pigs at slaughter can be used to assess the likely SD status of the source herds [9]. This proposition was tested in the current study by following antibody levels in the experimental pigs for 10 weeks after experimental infection.

The subsequent aims of this study were to investigate two other potential applications of serological assays, as well as comparing the utility of the Bhlp29.7 and whole cell ELISAs detecting either IgG or IgM. Being a serogroup-specific antigen, LPS is not considered suitable for development of a universal diagnostic tool [5,11,12], and therefore it was not included in the present evaluation. The first application involved monitoring antibody profiles in growing pigs in an infected herd to assess the herd infection dynamics and predict the time of infection, and the second explored using the ELISAs on muscle fluid ("meat juice") samples taken from pigs at slaughter. Sampling muscle at the abattoir avoids the biosecurity risks of on-farm visits, and muscle is generally easier to collect and process at slaughter than is blood. Hence muscle juice could be a convenient source of antibodies for routine screening of herds for evidence of infection with *B. hyodysenteriae*.

## Results

### Experimental infection

**Figure 1:**
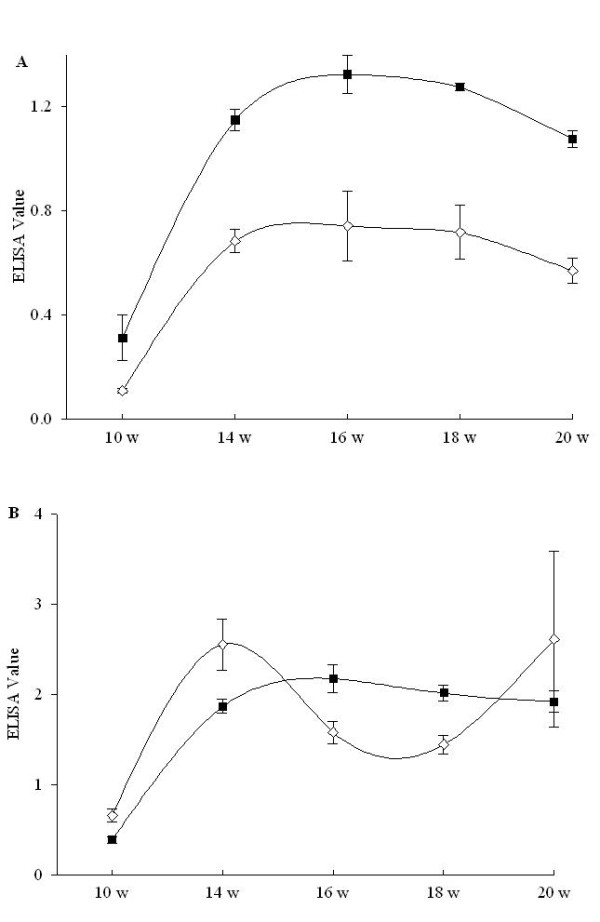
**Serum IgG (diamond symbols) and IgM (square symbols) responses of the pigs experimentally infected with *B. hyodysenteriae *and monitored over a 10 week period post-infection**. Panel A, ELISA results using whole cell plate-coating antigen; panel B, ELISA results using recombinant Bhlp29.7 as plate-coating antigen. ELISA Value represents the raw data of optical density. Data are the means ± SD.

Both experimentally challenged pigs excreted *B. hyodysenteriae *in their faeces in the week after challenge and showed transient diarrhoea without blood or mucus being present. They then recovered and remained healthy throughout the experimental period, and no subsequent spirochaete excretion was detected. IgG and IgM antibodies reacting with the WC antigen increased to a maximum at 4-6 weeks post-infection (PI); they then showed a slight decline, although they were still elevated at 10 weeks PI (Figure 1, panel A). In the case of the Bhlp29.7 antigen, antibody levels peaked at 4 weeks PI and the subsequent decline was more rapid than with the WC antigen, but the IgG level in one pig became elevated again after 8 weeks PI (Figure 1, panel B).

### Culture and PCR results in the cohort study

No pigs in the two cohorts were positive for *B. hyodysenteriae *by culture and PCR at any sampling, although non-pathogenic *B. innocens *and unidentified *Brachyspira *spp. were identified in the faeces of pigs from both farms at different times (Table 1). Two pigs from the cohort on farm C were positive for *B. pilosicoli *at 10 weeks of age. Two of the 24 pigs from farm C that were not part of the cohort were positive for *B. hyodysenteriae *by culture and PCR when sampled at the abattoir.

**Table 1:** Number of pigs positive for *Brachyspira *spp. using culture and PCR at five sampling periods in the cohort study on farms A and C

		Age in weeks				
		
Farm^a^		6	10	14	18	22
	*B. hyodysenteriae*	0	0	NT	0	0
A	*B. pilosicoli*	0	0	NT	0	0
(20 pigs)	*B. innocens*	0	8	NT	2	1
	Other *Brachyspira *spp.	1	4	NT	2	2

C	*B. hyodysenteriae*	0	0	0	0	0
(30 pigs)	*B. pilosicoli*	0	2	0	0	0
	*B. innocens*	0	0	4	3	0
	Other *Brachyspira *spp.	9	6	1	1	3

^a^Farm A free of infection with *B. hyodysenteriae*. Farm C infected with *B. hyodysenteriae*; NT, not tested

### Antibody profiles in the cohort study

**Figure 2:**
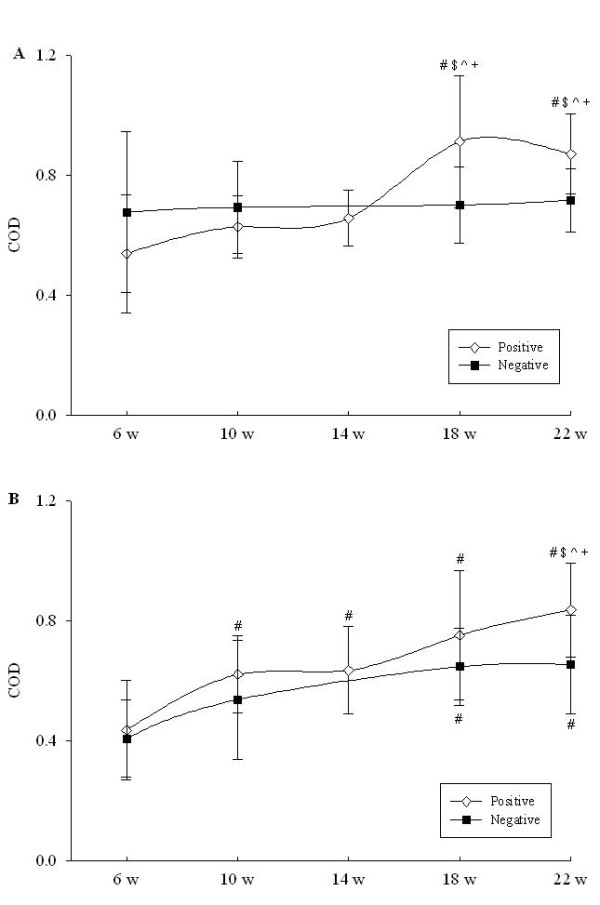
**Serum IgG concentrations in cohorts of pigs from uninfected farm A (square symbols) and infected farm C (diamond symbols) from six weeks to slaughter**. Panel A, ELISA results using whole cell plate coating antigen; panel B, ELISA results using recombinant Bhlp29.7 as plate coating antigen. ^# $ ^ ^indicate p < 0.05 compared with 6 w, 10 w and 14 w within the same group respectively, while ^+ ^indicates p < 0.001 compared to the same time point in the negative herd. COD represents calibrated optical density. Data are the means ± SD.

The average IgG response to the *B. hyodysenteriae *WC antigen in the 20 pigs on the uninfected farm A did not show a significant change during the course of experiment, while there was a highly significant (*P *< 0.0001) increase with time in the cohort on infected farm C (Figure 2, panel A). In the latter farm there was a steady increase in mean IgG from 6 weeks, with a peak at 18 weeks, followed by a slight decrease by 22 weeks. The pigs in farm C had significantly higher IgG levels than those in farm A at 18 and 22 weeks (*P *< 0.0001). Using Bhlp29.7 as the coating antigen, IgG levels in both cohorts showed a significant increasing trend (*P *< 0.0001), with a peak at 22 weeks (Figure 2, panel B). Mean IgG levels were higher in the pigs in the cohort on farm C than those from the cohort on farm A, and this difference was significant at 22 weeks (*P *< 0.001).

**Figure 3:**
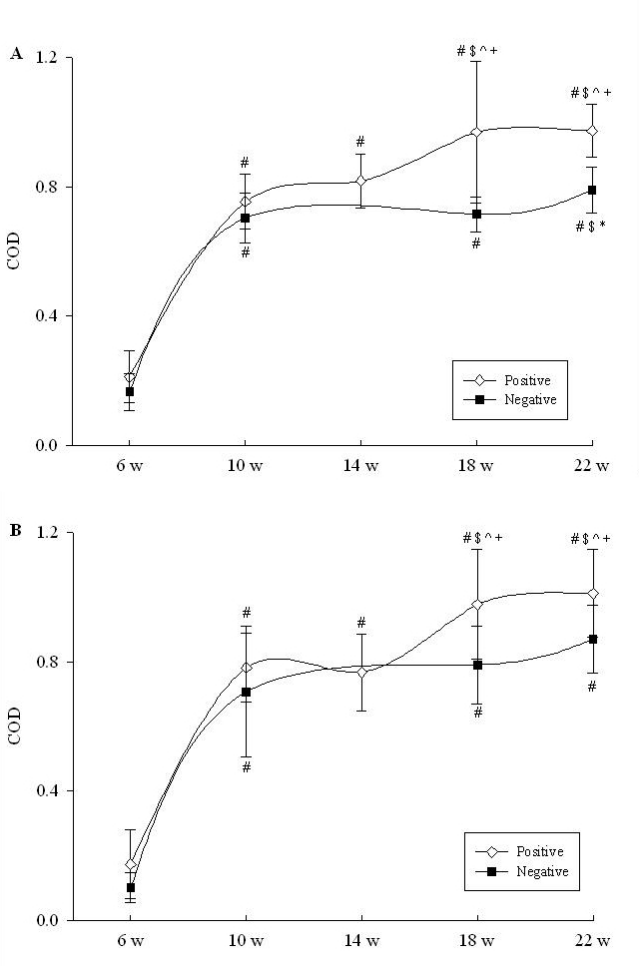
**Serum IgM concentrations in cohorts of pigs from uninfected farm A (square symbols) and infected farm C (diamond symbols) from six weeks to slaughter**. Panel A, ELISA results using whole cell plate coating antigen; panel B, ELISA results using recombinant Bhlp29.7 as plate coating antigen. ^# $ ^ ^indicate p < 0.05 compared with 6 w, 10 w and 14 w within the same group respectively, while ^+ ^indicates p < 0.001 compared to the same time point in the negative herd. COD represents calibrated optical density. Data are the means ± SD.

Unlike IgG dynamics, particularly in response to WC antigen, IgM antibody levels displayed a similar trend for both negative and positive herds regardless of whether WC or Bhlp29.7 were used as ELISA antigens (Figure 3). The average IgM levels in the cohort of pigs in both herds were elevated significantly at 10 week, and thereafter remained elevated compared to those at 6 week (*P *< 0.001).

### Cross-reactivities

**Figure 4:**
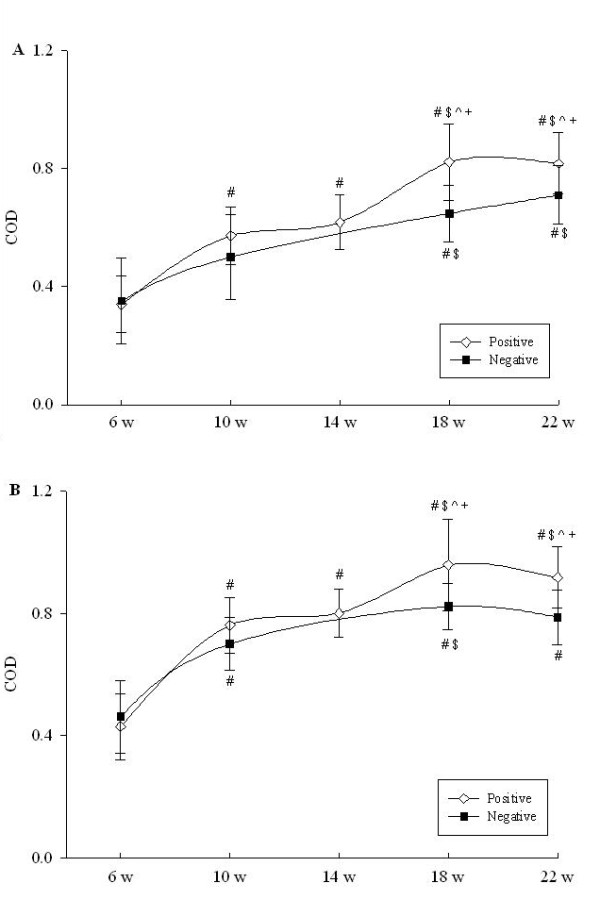
**Serum IgG concentrations in cohorts of pigs from uninfected farm A (square symbols) and infected farm C (diamond symbols) from six weeks to slaughter**. Panel A, ELISA results using *B. pilosicoli *whole cell plate coating antigen; panel B, ELISA results using *B. innocens *whole-cell antigen. ^# $ ^ ^indicate p < 0.05 compared with 6 w, 10 w and 14 w within the same group respectively, while ^+ ^indicates p < 0.001 compared to the same time point in the negative herd. COD represents calibrated optical density. Data are the means ± SD.

Using WC preparations of *B. pilosicoli *and *B. innocens *respectively as plate-coating antigens, in the cohorts from both farms a similar pattern of gradual and highly significant (*P *< 0.0001) increases in IgG levels to both preparations was observed from 6 to 22 weeks (Figure 4, panels A and B). For both preparations mean antibody levels were higher in the cohort of pigs from farm C than in those from farm A, and for both the differences were significant at 18 and 22 weeks (*P *< 0.001).

### Diagnostic threshold values in slaughtered pigs

The diagnostic threshold values for the slaughtered pigs, established using 100 serum or meat juice samples from pigs on each of the two non-infected farms, and calculated as three standard deviations above the mean absorbance value, are presented in Table 2.

**Table 2:** Mean and standard deviation of optical density readings for 100 serum samples from two non-infected herds, together with the calculated cut-off value for negativity by ELISA using either whole cell (WC) or recombinant Bhlp29.7 as plate coating antigens with serum and meat juice samples

	Serum				Meat juice			
	
	IgG		IgM		IgG		IgM	
	
	WC	Bhlp29.7	WC	Bhlp29.7	WC	Bhlp29.7	WC	Bhlp29.7
Mean ± SD	0.63 ± 0.1	0.52 ± 0.10	0.68 ± 0.11	0.78 ± 0.09	0.32 ± 0.06	0.68 ± 0.13	0.71 ± 0.09	0.64 ± 0.12
Cut-off value	0.93	0.83	1.0	1.06	0.49	1.08	0.99	0.96

### Sensitivity and specificity of ELISAs as a herd test

The numbers of pigs that gave positive results in the eight assays are shown in Table 3. Generally, at the herd level all these assays worked well, with five of the assays having both 100% specificity and sensitivity. Assays based on meat juice had 100% specificity and equivalent sensitivity to those based on serum. Only the WC IgM ELISA with serum lacked specificity, because a sample from uninfected herd A gave a positive reading. The Bhlp29.7 IgG ELISA with serum and the WC IgM ELISA with meat juice lacked 100% sensitivity, because in both cases none of the samples from infected herd D gave positive readings.

**Table 3:** Comparison of the number of pigs in five herds found positive by ELISA using either whole cell (WC) or recombinant Bhlp29.7 as plate coating antigens with serum and meat juice samples, and specificity and sensitivity at the herd level

	Serum				Meat juice			
	
	IgG		IgM		IgG		IgM	
	
Herd *^a^*	WC	Bhlp29.7	WC	Bhlp29.7	WC	Bhlp29.7	WC	Bhlp29.7
A	0	0	1	0	0	0	0	0
B	0	0	0	0	0	0	0	0
C	1	1	2	2	3	1	4	1
D	2	0	1	1	1	1	0	1
E	2	1	4	2	3	1	3	1
Total positive in herds C-E	5	2	7	5	7	3	7	3
Specificity (%)	100	100	50	100	100	100	100	100
Sensitivity (%)	100	66.7	100	100	100	100	66.7	100

*^a ^*Herds A and B known not to be infected with *B. hyodysenteriae*. Fifty pigs were sampled from each herd.

### Detection levels

All four WC systems detected more positive serum samples in the three infected herd than did the corresponding Bhlp29.7 systems (ranging from 5 to 7 positives compared to 2 to 5, respectively). The four tests using serum between them yielded 19 positive samples from the positive herds, compared to 20 using meat juice. Overall, however, only eight individual serum samples were positive compared to 13 meat juice samples.

## Discussion

The experimental infection study demonstrated that pigs challenged with *B. hyodysenteriae *developed elevated concentrations of IgG and IgM in their serum to both WC and Bhlp29.7 antigen preparations. Antibody levels had a dramatic increase 4-6 weeks after infection, and hence this might be an optimal interval after infection to seek serological evidence of infection using these antigens. Thereafter the IgG levels to Bhlp29.7 decreased more rapidly than did those to WC. One of the pigs showed a secondary increase in Bhlp29.7-reactive IgG between 18 weeks and slaughter at 22 weeks, suggesting that a proliferation of the spirochaete may have occurred in that pig at around 18 weeks of age. Unexpectedly, an increase in antibody was not detected in this pig using the WC ELISA. As antibody levels to both antigen preparations remained elevated from 10 to 22 weeks, this confirms that sampling pigs at slaughter age should detect elevated antibody levels to the spirochaete.

In the cohort study, at no time were any of the sampled pigs found to be positive for *B. hyodysenteriae *by using culture and PCR. This was despite farm C being known to have SD, and having two colonized pigs detected amongst the additional 24 pigs from that farm that were sampled at the abattoir. There was no overt disease being expressed at farm C during the time of the study, and presumably the extent of colonization in individual pigs also was low. *B. pilosicoli*, *B. innocens *and/or other *Brachyspira *spp. were identified at different times in some pigs on both farms. This observation demonstrated that the culture methods used for detection were appropriate, but also indicated that the pulsed medication at farm C did not completely prevent colonization with *Brachyspira *species. Without careful veterinary surveillance of this farm, it would be difficult to recognize it as being infected with *B. hyodysenteriae*. Despite the lack of culture and PCR evidence for colonization, antibody levels to *B. hyodysenteriae *increased with time on farm C, to peak at around 18 weeks of age. Based on the experimental infection study, this suggests that there was a degree of spirochaete proliferation amongst the cohort of pigs on farm C at around 12-14 weeks of age. This sort of information could be helpful for the consulting veterinarian, who might decide to medicate the pigs at this time to prevent excessive proliferation of the spirochaete that otherwise could depress growth rates and affect feed conversion.

Differences were observed between the IgG response patterns using WC and Bhlp29.7 as plate-coating antigens. *B. hyodysenteriae *WC preparations have been thought to potentially generate false positive results arising from cross-reactivities associated with exposure to other *Brachyspira *species, or other bacteria with cross-reactive proteins [5,7]. In the pigs from the cohort on farm A, where SD did not occur, there was no significant temporal change in IgG levels to the *B. hyodysenteriae *WC preparation, but there were significant increases to WC preparations from both *B. pilosicoli *and *B. innocens*. The seroconversions in responses to the protein content of *B. pilosicoli *and *B. innocens *further confirmed the presence of these organisms in both farms, in accordance with the results of culture and PCR. Most importantly, the data provided additional evidence supporting the specificity of the *B. hyodysenteriae *WC IgG ELISA as a diagnostic test. On the other hand, it is possible that exposure to *B. hyodysenteriae *may generate antibodies that cross-react with WC preparations of *B. pilosicoli *and *B. innocens*. This was because antibody levels to antigen preparations from the latter two species were higher in the pigs from farm C than in those from farm A, despite these spirochaetes being present on both farms; moreover, their pattern of increase, peaking at around 18 weeks on farm C, closely resembled the pattern seen with the *B. hyodysenteriae *WC preparation.

Although IgG antibody levels to the *B. hyodysenteriae *WC preparation and to Bhlp29.7 both gave evidence of infection amongst the cohort on farm C, and could be used as tools to monitor infection, the WC preparation gave a better discrimination between the two cohorts at 18 weeks. An apparent problem with the Bhlp29.7 ELISA in this study was the significant temporal increase in antibody levels in the pigs from farm A, reducing the discrimination between results for the two sets of sera. This is paradoxical, given that the use of specific recombinant antigens like Bhlp29.7 should reduce potential cross-reactivity [5]. However, the gene for Bhp29.7 has been identified in *B. innocens *strain B256^T [13]^, and the presence of such strains might generate false positive cross-reactivities and reduce the discriminatory power of this ELISA. Even more problematic is the recent report that the gene encoding Bhlp29.7 could not be amplified from 33 (45%) recent German isolates of *B. hyodysenteriae *[14]. Although recently Lobova et al reported that Immunoblotting using Bhlp29.7 in conjunction with culturing method was a valuable tool for detecting swine herds latently infected with *B. hyodysenteria *[15], the specificity of the Bhlp29.7 is still not clear. Hence further work is required to identify alternative antigenic surface exposed proteins specific to *B. hyodysenteriae *that can be used in serological assays. Recombinant forms of such proteins are useful as plate-coating antigens as they are easier to prepare as a standard product than are WC preparations.

The change in IgM also demonstrated great magnitude in the experimental animals after exposure to *B. hyodysenteriae*. The performance of the IgM ELISA systems based on WC and Bhlp29.7 was then evaluated using the field samples in the cohort study. However, IgM levels to both antigens demonstrated a significant increase at 10 week on farm A, indicating that IgM is less specific than IgG in providing evidence of exposure to the spirochaete.

To further explore the suitability of ELISA systems using WC and Bhlp29.7 as herd tests, swine sera were collected from the herds with high health status and herds with a history of SD. A sample size of 50 animals within each herd was chosen to achieve an appropriate confidence of 95% of detecting an individual infected pig based on the previous study [9,16]. Due to considerable variation in antibody response observed in the non-infected pigs, the value of three standard deviations above the mean was applied as diagnostic thresholds. Although the high stringent cut-off value (mean + 3 SD) might prioritize the test specificity and compromise the test sensitivity, the preliminary study in a clinical setting demonstrated that the assays based on both antigens could discriminate positive herds from negative herds. Certainly testing more herds is required to accurately estimate the assay performance with more reliable cut-off established from a large population of non-infected herds in the future.

Additionally meat juice has been reported to be a useful alternative to serum as a source of antibodies, and has been used in a number of sero-epidemiological studies and in surveillance for porcine infections [17-21]. A comparative study on serum and meat juice samples from the same source of herds was undertaken using the ELISA systems described above. The assays based on meat juice achieved equivalent sensitivity and specificity as serum specimens (Table 3). It was recognized that the current study could be strengthened by including more samples from negative and positive farms, but nevertheless it was clear from the results that meat juice from some pigs on the infected farms had elevated IgG and IgM levels to *B. hyodysenteriae*. This finding supported our hypothesis that testing meat juice for specific antibodies could be used as a surveillance tool to help detect farms infected with *B. hyodysenteriae*. The noteworthy, in both serum and meat juice tests, the IgG WC ELISA had 100% sensitivity and specificity as a herd test and gave a high detection rate. This finding further confirmed the suitability of using a IgG ELISA coated with *B. hyodysenteriae *WC antigen as an aid in the diagnosis of infected herds.

## Conclusions

Based on the three independent studies, IgG ELISAs using either WC or Bhlp29.7 as plate-coating antigens were shown to be useful for monitoring the dynamics of *B. hyodysenteriae *infection in grower pigs. Of the two antigens, the WC preparation tended to give better discrimination between pigs from infected and non-infected farms. As recombinant proteins are easier to produce and standardize than are WC preparations, it would be useful to identify and evaluate additional immunogenic surface proteins of *B. hyodysenteriae *as ELISA antigens. Meat juice samples collected from pigs on infected farms were shown to contain specific antibodies to *B. hyodysenteriae*, and analysis of this material could be incorporated into routine health surveillance. However, the present study should be considered as a preliminary one and further investigation is required to confirm the findings, particularly with a large number of samples.

## Methods

### Permissions

The study was conducted with the approval of the Murdoch University Animals Ethics Committee. The authors received consent from the pig owners to use their pigs in this study.

### Experimental infection

Two ten-week-old pigs from a high health status farm, where no evidence of SD had ever been recorded (farm A) were housed in an isolation animal house and experimentally challenged by stomach tube with cultures containing 10^10 ^cells of *B. hyodysenteriae *strain WA1 over three successive days, as previously described [22]. Blood samples were collected pre-infection, then at 4, 6, 8 and 10 weeks post-infection. The health of the animals was monitored daily and faecal samples were collected every 2-3 days for the first two weeks and then at the same time as the blood samples.

### Cohort study

Fifty pigs born at farm A were divided into a cohort group of 20 that remained at farm A and 30 that were transferred to farm C after weaning. Farm C had a previous diagnosis of SD based on clinical signs, post-mortem examination, and faecal culture and PCR of *B. hyodysenteriae*. Although farm C remained infected, clinical signs in grower pigs were suppressed using routine in-feed medication with zinc bacitracin at 270 mg/kg of feed, pulsed week-on week-off from 10 weeks of age.

The pigs were ear-tagged and pairs of blood and faecal samples were collected at 6 weeks of age and thereafter every 4 weeks until slaughter at 22 weeks. One collection from farm A was missed at 14 weeks, and two and six samples were missed from farm C at 18 and 22 weeks respectively due to ear tag loss or dying of unknown diseases. Additional faecal samples were collected at the abattoir from 24 other pigs from farm C at the same time the pigs in the cohort were slaughtered.

### Meat juice and serum samples

Fifty sections of diaphragm and sera were collected from pigs from each of five farms, including farms A and C where the cohort study was conducted, following their slaughter at a local abattoir. All pigs were healthy and no abnormalities were found in the carcasses. Farms A and B were of high health status and had never had evidence of SD. Farms C, D and E all had previous diagnoses of SD based on clinical signs, post-mortem examination, and faecal culture and PCR for *B. hyodysenteriae*.

### Detection of *Brachyspira *species using culture and PCR

The faecal samples were subjected to selective anaerobic culture for *Brachyspira *species [23]. Growth on the plates was tested by PCR for *B. hyodysenteriae *and *Brachyspira pilosicoli *[3], and for *Brachyspira innocens *and general *Brachyspira *spp. using the method of Weissenbock et al [24].

### Serum and meat juice collection

For experimentally infected pigs and the pigs in cohorts that were repeatedly sampled, the animals were manually restrained and blood was obtained from the anterior vena cava using a vacutainer and 20-gauge needle. The final collection from the pigs in the cohort occurred at the abattoir. The blood was allowed to stand overnight and the serum was removed, mixed with an equal volume of 100% glycerol and stored at -20°C. Where appropriate, faecal samples from the same pigs were collected for culture and PCR.

The method of Nielsen et al [18] was used for obtaining muscle fluid from pigs killed at the abattoir. Briefly, a 3 × 1 × 1 cm piece of muscle was excised from the diaphragm and placed into the mouth of a small sterile plastic funnel over a sterile bijou bottle; this was covered with plastic food-wrap and held at -20°C overnight. After thawing, the muscle fluid ("meat juice") that was passively released was collected and stored at -20°C.

### Preparation of ELISA antigens

Whole-cell (WC) antigen preparations and recombinant Bhlp29.7 were used as plate-coating antigens in indirect ELISAs to detect antibodies in porcine serum and meat juice. WC antigens were prepared from cultures of *B. hyodysenteriae *strain WA1, *B. pilosicoli *strain 95/1000 and *B. innocens *strain B256^T ^respectively, using the sonication method described by Wright et al [7]. Recombinant Bhlp29.7 was expressed and purified as a 6 × His tag fusion protein in *Escherichia coli*, as described previously [9,22]. Protein concentrations were determined using the Bradford protein assay (Biorad, USA).

### ELISA procedures

The wells in 96-well microtitre plates (Immulon 4HBX, Dynex Technologies) were coated either with 100 μl of a WC preparation of the *Brachyspira *species (2 μg/ml) or purified Bhlp29.7 recombinant protein (3 μg/ml; [3], 2009) in 0.1 M carbonate buffer (pH 9.6) at 4°C overnight. The wells were blocked with 3% skim-milk powder in phosphate buffered saline (PBS: pH 7.2) for 1 h, then washed three times with PBS containing 0.05% Tween 20. Serum samples that were diluted 1:400 for the WC ELISA and 1:200 for the Bhlp29.7 ELISA, or meat juice samples diluted 1:20, were added to the wells of the plates and incubated for 2 h at room temperature (RT). Each sample was assayed in triplicate and the mean value used. After washing as described above, goat anti-swine lgG (1:10,000, KPL) or lgM (1:30,000, AbD Serotec) horseradish peroxidase conjugates was added to each well and incubated for 1 h at RT on a rocking platform. Colour development was initiated by adding 3,3',5,5'-tetramethyl-benzidine liquid substrate (Sigma, USA) and was stopped after 15 min by adding 0.5 M sulphuric acid. The optical density (OD) was measured at 450 nm on a microplate reader (Biorad Model 3550-UV).

For across-plate standardization, positive controls were added in triplicate to each plate. These were obtained from the Reference Centre for Intestinal Spirochaetes at Murdoch University and consisted of hyperimmune sera produced in three 14-week-old experimental pigs by vaccinating them twice intramuscularly at a three-week interval with formalinized bacterins from *B. hyodysenteriae *B78^T^, *B. pilosicoli *strain 1648 or *B. innocens *strain B256^T^, respectively, in Freund's incomplete adjuvant. The positive meat juice sample control was collected from a pig experimentally infected with *B. hyodysenteriae *and killed after developing clinical signs of SD. Calibrated ODs (COD) were calculated according to the following formula: average of (OD value of test sample - OD value of blank control)/(OD value of control sample - OD value of blank control).

### Data analysis

The data were analyzed using SPSS 16.0 for Windows XP™ and Sigmaplot (version 11.0, Systat Software Inc, San Jose, USA). For the cohort study, differences within a group with time were analysed by one-way analysis of variance with individual pairs of results compared using the Tukey-Kramer Multiple Comparisons Test. Comparisons between the two groups in the cohort study at each time point were assessed using the Student's two-tailed *t*-test for normally distributed data and Mann-Whitney test for non-normally distributed data, with significance accepted at the 5% level. Data are presented as mean ± standard deviation (SD). In the evaluation of ELISA performance with serum and meat juice samples, the cut-off value for negativity in the assays was established based on the mean ELISA reading plus three standard deviations from the 100 samples from each of the two uninfected farms. The presence of one or more ELISA result above this value from a farm was taken to indicate that the assay had identified the farm as being infected.

## Authors' contributions

YS and DJH conceived and designed the study, prepared the paper and analyzed the data. YS performed the laboratory work. YS and BF conducted the animal work and collected the samples. All authors read and approved the final manuscript.

## References

[B1] Hampson DJ Hampson DJ, Fellström C, Thomson JR Diseases of Swine Swine dysentery 2006 Chapter 48 Oxford, UK Blackwell Publishing 785 805

[B2] Song Y Hampson DJ Development of a multiplex qPCR for detection and quantitation of pathogenic intestinal spirochaetes in the faeces of pigs and chickens Vet Microbiol 2009 137 1-2 129 136 10.1016/j.vetmic.2008.12.020 19171443

[B3] La T Phillips ND Hampson DJ Development of a duplex PCR assay for detection of *Brachyspira hyodysenteriae *and *Brachyspira pilosicoli *in pig feces J Clin Microbiol 2003 41 7 3372 3375 10.1128/JCM.41.7.3372-3375.2003 12843096 PMC165297

[B4] Atyeo RF Oxberry SL Combs BG Hampson DJ Development and evaluation of polymerase chain reaction tests as an aid to diagnosis of swine dysentery and intestinal spirochaetosis Lett Appl Microbiol 1998 26 2 126 130 10.1046/j.1472-765X.1998.00294.x 9569695

[B5] La T Hampson DJ Serologic detection of *Brachyspira *(*Serpulina*) *hyodysenteriae *infections Anim Health Res Rev 2001 2 1 45 52 11708746

[B6] Smith SC Barrett LM Muir T Christopher WL Coloe PJ Application and evaluation of enzyme-linked immunosorbent assay and immunoblotting for detection of antibodies to *Treponema hyodysenteriae *in swine Epidemiol Infect 1991 107 2 285 296 10.1017/S0950268800048937 1936151 PMC2272052

[B7] Wright JC Wilt GR Reed RB Powe TA Use of an enzyme-linked immunosorbent assay for detection of *Treponema hyodysenteriae *infection in swine J Clin Microbiol 1989 27 3 411 416 2715317 10.1128/jcm.27.3.411-416.1989 PMC267331

[B8] Joens LA Nord NA Kinyon JM Egan IT Enzyme-linked immunosorbent assay for detection of antibody to *Treponema hyodysenteriae *antigens J Clin Microbiol 1982 15 2 249 252 7040447 10.1128/jcm.15.2.249-252.1982 PMC272070

[B9] La T Phillips ND Hampson DJ Evaluation of recombinant Bhlp29.7 as an ELISA antigen for detecting pig herds with swine dysentery Vet Microbiol 2009 133 1-2 98 104 10.1016/j.vetmic.2008.06.003 18619744

[B10] Fisher LF Olander HJ Shedding of *Treponema hyodysenteriae*, transmission of disease, and agglutinin response to pigs convalescent from swine dysentery Am J Vet Res 1981 42 3 450 455 7271009

[B11] Hampson DJ Maltas CD Stephens CP McKechnie K Buller NB Serogroups of Australian isolates of *Serpulina hyodysenteriae* Aust Vet J 1994 71 10 347 10.1111/j.1751-0813.1994.tb00918.x 7848186

[B12] Hampson DJ Mhoma JR Combs BG Lee JI Serological grouping of *Treponema hyodysenteriae* Epidemiol Infect 1990 105 1 79 85 10.1017/S0950268800047671 2116974 PMC2271794

[B13] La T Tan P Phillips ND Hampson DJ The distribution of *bmpB*, a gene encoding a 29.7 kDa lipoprotein with homology to MetQ, in *Brachyspira hyodysenteriae *and related species Vet Microbiol 2005 107 3-4 249 256 10.1016/j.vetmic.2005.01.016 15863284

[B14] Barth S Richter M Herbst W Virulence and fitness gene patterns in German *Brachyspira hyodysenteriae *field isolates Proc 5th Int Conf Colonic Infect Anim and Humans 2009 León, Spain 31

[B15] Lobova D Prasek J Cizek A Celer V Evaluation of the use of recombinant Bhlp29.7 in immunoblotting with pig serum as a means to identify herds infected with *Brachyspira hyodysenteriae* Lett Appl Microbiol 2011 Oct 53 4 466 72 21838749 10.1111/j.1472-765X.2011.03134.x

[B16] Mhoma JR Hampson DJ Robertson ID A serological survey to determine the prevalence of infection with *Treponema hyodysenteriae *in Western Australia Aust Vet J 1992 69 4 81 84 10.1111/j.1751-0813.1992.tb15555.x 1605788

[B17] Le Potier MF Fournier A Houdayer C Hutet E Auvigne V Hery D Sanaa M Toma B Use of muscle exudates for the detection of anti-gE antibodies to Aujeszky's disease virus Vet Rec 1998 143 14 385 387 10.1136/vr.143.14.385 9802195

[B18] Nielsen B Ekeroth L Bager F Lind P Use of muscle fluid as a source of antibodies for serologic detection of *Salmonella *infection in slaughter pig herds J Vet Diagn Invest 1998 10 2 158 163 10.1177/104063879801000207 9576343

[B19] Mortensen S Strandbygaard B Botner A Feld N Willeberg P Monitoring porcine reproductive and respiratory syndrome virus infection status in swine herds based on analysis of antibodies in meat juice samples Vet Res 2001 32 5 441 453 10.1051/vetres:2001136 11592614

[B20] Nowak B von Muffling T Chaunchom S Hartung J *Salmonella *contamination in pigs at slaughter and on the farm: a field study using an antibody ELISA test and a PCR technique Int J Food Microbiol 2007 115 3 259 267 10.1016/j.ijfoodmicro.2006.10.045 17292500

[B21] Beck R Gaspar A Mihaljevic Z Marinculic A Stojcevic D Brstilo M Evaluation of ELISA for detection of *Trichinella *antibodies in muscle juice samples of naturally infected pigs Vet Parasitol 2005 132 1-2 91 95 10.1016/j.vetpar.2005.05.034 15993544

[B22] La T Phillips ND Reichel MP Hampson DJ Protection of pigs from swine dysentery by vaccination with recombinant BmpB, a 29.7 kDa outer-membrane lipoprotein of *Brachyspira hyodysenteriae* Vet Microbiol 2004 102 1-2 97 109 10.1016/j.vetmic.2004.06.004 15288932

[B23] Jenkinson SR Wingar CR Selective medium for the isolation of *Treponema hyodysenteriae* Vet Rec 1981 109 17 384 385 10.1136/vr.109.17.384 7340069

[B24] Weissenbock H Maderner A Herzog AM Lussy H Nowotny N Amplification and sequencing of *Brachyspira *spp. specific portions of nox using paraffin-embedded tissue samples from clinical colitis in Austrian pigs shows frequent solitary presence of *Brachyspira murdochii* Vet Microbiol 2005 111 1-2 67 75 10.1016/j.vetmic.2005.09.002 16213113

